# Supplementary material for: A Novel KRAS Antibody Highlights a Regulation Mechanism of Post-Translational Modifications of KRAS during Tumorigenesis
Source: Int J Mol Sci. 2020 Sep 2;21(17):6361. doi: 10.3390/ijms21176361 (PMC7504708; doi:10.3390/ijms21176361)
Supplement: Supplementary file 1 [file ijms-21-06361-s001.pdf]

Supplementary Figures (Assi et al.)

A novel KRAS antibody highlights a regulation mechanism of post-translational modifications of KRAS during tumorigenesis

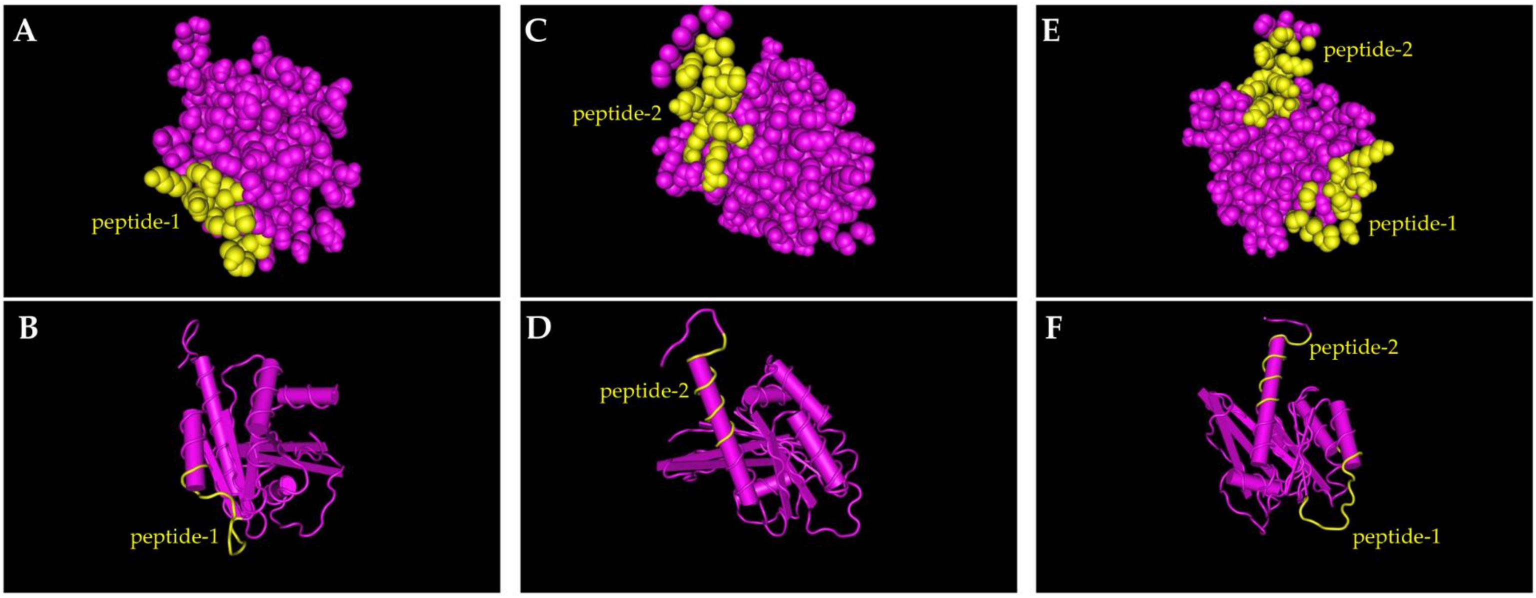

Figure S1. ***In silico*** **analysis of peptide location in 3D structure of KRAS.** The KRAS 4DSU crystallography structure was obtained from Protein Data Bank. The software Cn3D was used to highlight peptide location. Panels **A**, **C** and **E** represent a space-filling structure of KRAS and panels **B**, **D** and **F** show a cartoon representation of the crystal structure of KRAS illustrating the location of α-helices (cylinders) and β-sheets (rectangles). (**A-B**) Peptide-1 (in yellow) is present in the backbone outside of α-helix and β-sheet structures; the location of peptide-1 indicates that it is exposed and, therefore, accessible for an antibody. (**C-D**) Peptide-2 (in yellow) is mainly present in α-helix structure; its location suggests that it is probably accessible for an antibody. (**E-F**) Pictures showing the presence of both peptide-1 and peptide-2 on KRAS structure.

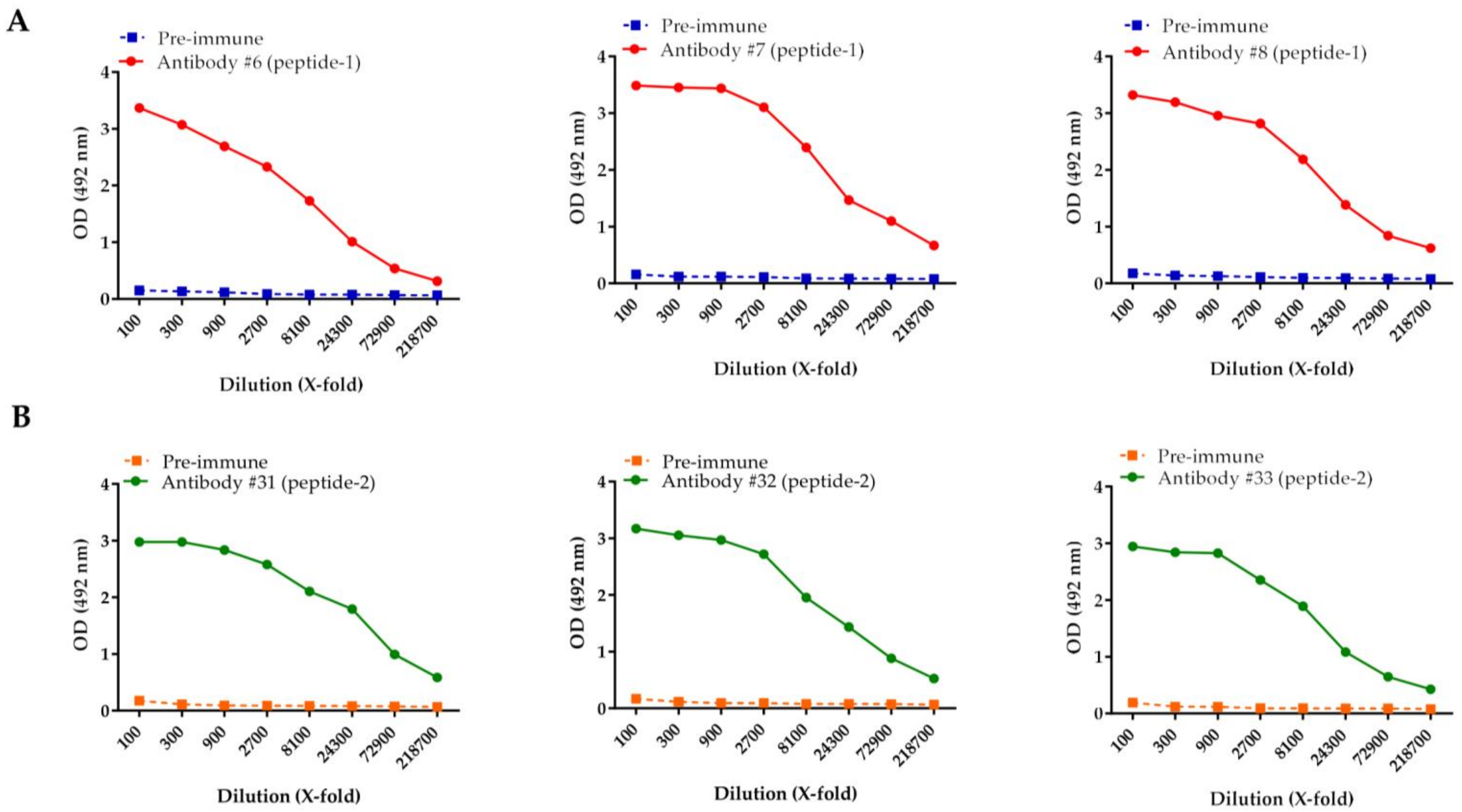

Figure S2. Immune response of different rabbits injected with peptide-1 and peptide-2. **(A)** ELISA with pre-immune sera, antibody #6, antibody #7 and antibody #8 on wells coated with peptide-1. **(B)** ELISA with pre-immune sera, antibody #31, antibody #32 and antibody #33 on wells coated with peptide-2.

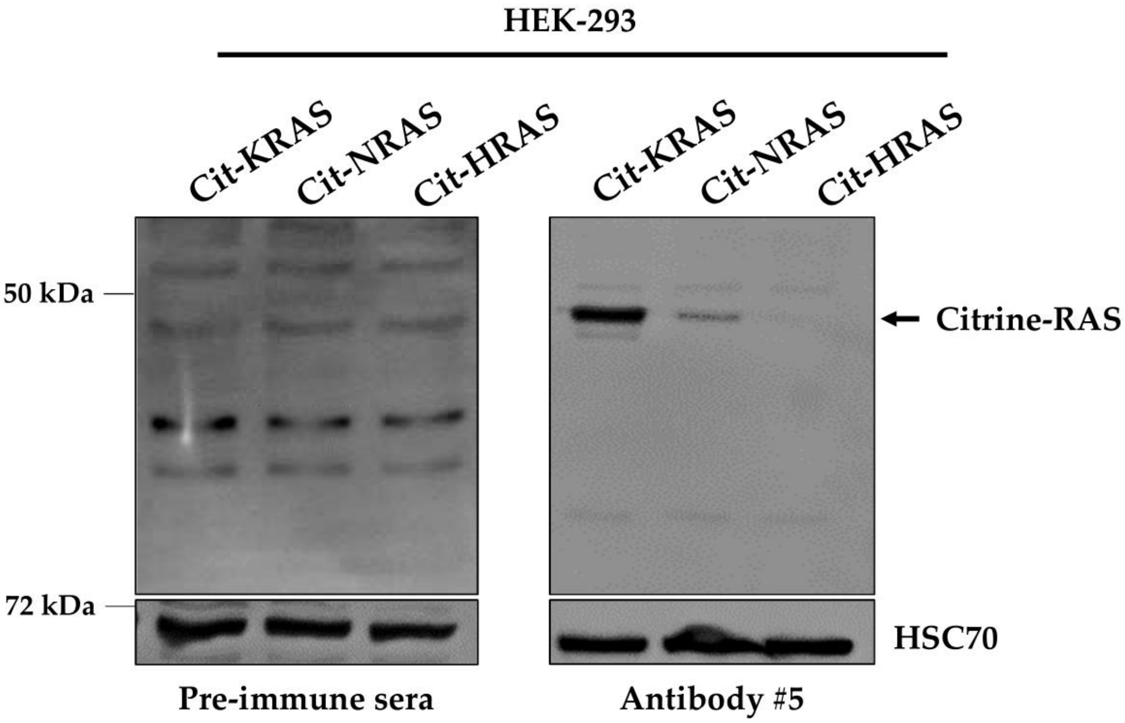

Figure S3. Specificity control for antibody #5. Western blot on protein lysates from HEK-293 cells transfected with the different citrine-fused RAS plasmids. Membranes were blotted with pre-immune serum from rabbit #5 or antibody #5. Data show no specific detection for citrine-RAS fusion proteins with pre-immune serum, while citrine-KRAS fusion protein was sensitively detected by antibody #5.
